# STEAP: Camera-Based Longitudinal Classroom Behavior Sensing and Static–Temporal Data Fusion for Academic Performance Prediction in Software Engineering Education

**DOI:** 10.3390/s26175677

**Published:** 2026-09-07

**Authors:** Jialing Wang, Qikai Lin, Yunhong Ding, Jingyu Liu, Bo Qi

**Affiliations:** 1 School of Computer Science and Information Engineering, Harbin Normal University, No. 1 Shida Road, Hulan District, Harbin 150025, China; 2024300731@stu.hrbnu.edu.cn (J.W.); 2024300716@stu.hrbnu.edu.cn (Q.L.); 2 School of Astronautics, Harbin Institute of Technology, Harbin 150001, China; 22b918100@stu.hit.edu.cn

**Keywords:** camera-based sensing, classroom behavior analysis, longitudinal learning analytics, academic performance prediction, static–temporal data fusion, multimodal learning analytics

## Abstract

Predicting academic performance in face-to-face computing and software engineering courses is hindered by the limited availability of fine-grained process data. This study proposes STEAP, a camera-based static–temporal fusion framework that integrates longitudinal classroom behavior sensing with conventional educational records. Classroom videos from 375 undergraduates enrolled in four computing-related courses were collected over nine teaching weeks. Camera-derived observable behaviors were organized into student-level weekly sequences and transformed into outcome-independent longitudinal representations. Multiple machine-learning classifiers were subsequently applied to predict students’ academic performance. Checkpoint-specific predictions were conducted at Weeks 3, 6, and 9, with each prediction using only the classroom behavioral information available up to the corresponding time point. Using the complete nine-week Temporal representation together with the pre-course Background variables, XGBoost achieved the strongest classification performance among the evaluated models, with an Accuracy of 0.867, a Macro F1 of 0.862, and an At-risk Recall of 0.924. The checkpoint analyses further indicated that classroom behavioral information collected during the early course stage already provided useful predictive information without incorporating behavioral observations from subsequent weeks. After further integrating pre-course background variables and regular assessment information, the final fusion model achieved an Accuracy of 0.896 and a Macro F1 of 0.895. Overall, longitudinal camera-derived classroom behavior provides complementary predictive information beyond conventional educational information and supports the feasibility of earlier academic-risk identification at different course checkpoints.

## 1. Introduction

Predicting academic performance is an important task in computing and software engineering education, where students must progressively integrate conceptual knowledge, programming practice, problem solving, and project work. Educational data mining and learning analytics have provided a broad methodological basis for identifying patterns associated with academic outcomes and supporting instructional decision making [1,2]. However, prediction studies in higher education still rely mainly on demographic characteristics, prior achievement, assessment records, and learning-platform activity [3,4]. Machine-learning methods have also been widely applied to academic-performance and early-drop prediction [5]. Although learning management system traces can support accurate prediction, they primarily describe activity in digital environments [6]. For courses delivered mainly through face-to-face instruction, these data provide limited evidence of how students’ observable classroom behavior changes over time.

Camera-based classroom sensing offers a non-contact means of supplementing conventional educational records with process-level observations. Computer vision can transform classroom video into quantitative indicators of visible states, and previous work has demonstrated the feasibility of estimating student engagement-related behavior from classroom recordings [7]. Nevertheless, authentic classrooms present challenging detection conditions because students often occupy small image regions and are densely distributed, while desks, peers, camera angle, illumination, and seating distance introduce scale variation and partial occlusion. Student analysis in post-secondary classrooms has therefore been treated as a crowded small-object detection problem [8], and related studies have shown that density, multi-scale variation, and occlusion can substantially affect behavior recognition [9]. Cascade R-CNN is relevant to this setting because its progressively stricter intersection-over-union thresholds refine object localization across multiple stages [10]. However, a detected posture does not have a unique educational meaning; for example, a lowered head may reflect reading, note-taking, fatigue, or distraction. Because student engagement is multidimensional and context-dependent [11,12], camera-derived states should be interpreted as externally observable behavioral indicators rather than direct measures of attention, engagement, cognition, motivation, or learning quality.

Most classroom-video research has focused on behavior-recognition accuracy, attention-related state estimation, teaching evaluation, or detection architectures. Such work establishes whether visible states can be localized and classified, but detection metrics alone do not show whether these indicators contribute useful information to academic-outcome prediction. Process-oriented multimodal learning analytics has shown that observable behavioral evidence can support the interpretation of learning interactions [13], while systematic reviews indicate that multimodal data fusion can enrich learning-process analysis but also raises challenges involving alignment, interpretation, validation, and responsible use [14,15]. Yet it remains unclear whether longitudinal classroom-video representations provide predictive value beyond conventional educational information. A meaningful camera-based framework should therefore evaluate both detection reliability and the incremental contribution of video-derived behavioral representations.

The temporal organization of classroom behavior is also important. A single observation or course-level average may obscure changes in students’ visible activity over time. Time-series, sequence-classification, and progressive-temporal studies have shown that the timing and accumulation of learning behaviors can provide information beyond aggregate frequencies [16,17,18]. Temporal multimodal analyses likewise suggest that learning outcomes may be associated with how behavioral evidence unfolds over time [19], while longitudinal research indicates that classroom engagement and motivation may change across repeated observations [20]. These findings motivate representing classroom behavior as longitudinal trajectories and evaluating predictions at Weeks 3, 6, and 9 using only information available up to each checkpoint. Multi-source learning-analytics research further suggests that combining behavioral and structured educational data can improve prediction [21,22], although heterogeneous signals should not be treated as equivalent measurements of learning [23]. Structured educational information and observable classroom behavior should therefore retain their distinct meanings when their predictive contributions are evaluated.

Three research gaps motivate this study. First, the incremental predictive value of longitudinal camera-derived classroom behavior beyond conventional educational information remains insufficiently established. Second, behavioral representations used to evaluate this contribution should be constructed independently of the final academic outcome, because label-dependent feature construction can introduce target leakage and yield overly optimistic performance estimates [24]. Third, it remains unclear how early in a course classroom behavioral information becomes sufficiently informative for academic-risk prediction.

To address these gaps, this study proposes STEAP, a camera-based Static–Temporal Educational Analytics and Prediction framework. STEAP converts classroom video into longitudinal student-level observable-behavior sequences, constructs outcome-independent behavioral representations, and integrates them with conventional educational variables for academic-performance prediction. Checkpoint-specific analyses at Weeks 3, 6, and 9 further evaluate the incremental and time-bounded predictive value of classroom behavior. The study therefore aims to clarify the complementary role of longitudinal observable classroom behavior in academic-performance prediction and at-risk student identification.

## 2. Materials and Methods

### 2.1. Research Design, Participants, and Data Sources

This study adopted a longitudinal classroom-observation and machine-learning prediction design to examine whether camera-derived behavioral trajectories provide complementary predictive information beyond conventional educational information. The participants were 375 undergraduate students majoring in Software Engineering or Applied Statistics who were enrolled in four computing-related courses at a university in China. The observed sessions were theory-based classes in which laptop use was not permitted. Instruction was conducted primarily under conventional seated classroom conditions rather than movement-intensive laboratory or practical activities. Classroom observations were conducted over nine consecutive teaching weeks. To examine stage-specific variations in observable classroom behavior, Weeks 1–3 were defined as the early stage, Weeks 4–6 as the middle stage, and Weeks 7–9 as the late stage. Because the study combined classroom-video observations with educational records, informed consent, privacy protection, restricted data access, and appropriate limits on educational use were treated as core design requirements [25].

In this study, “static” educational information refers to structured student-level variables represented by a single categorical or numerical value rather than by a longitudinal sequence; the term does not imply that all such variables were available before the course. The student-level educational information used in this study included gender, major, entrance score, and regular assessment score. Gender and major characterized student background, entrance score represented prior academic preparation, and regular assessment score reflected course-related academic performance. The first three variables were available before the course, whereas the regular assessment score was generated during the course and used only in the final classification analysis. The regular assessment score did not include the final-examination score used to define the prediction target. Detailed information on variable type, domain, meaning, and availability is provided in Table 1.

### 2.2. Classroom Behavior Extraction and Temporal Feature Construction

Classroom videos were recorded using fixed-position cameras covering the main student seating areas without interfering with normal teaching activities. Because camera placement followed the layout of each classroom, viewing angles varied across recording sessions; the independent effect of camera angle on detection and classification performance was not separately evaluated in this study. Frames were sampled from the videos at 30 s intervals, producing an initial dataset of 3800 images. The 30 s interval was used to obtain standardized repeated observations across teaching weeks and was not intended to provide a comprehensive record of all classroom behaviors.

Behavior annotation. Three annotators used LabelMe [26] to manually annotate the screened classroom images. The behavioral categories were defined primarily from visually observable head-orientation and head-posture cues within the annotated regions. Specifically, LF represented looking toward the instructional area, LD represented an observable lowered-head or head-down state, and Other represented visible states that did not meet the predefined criteria for LF or LD. A cross-annotation procedure was adopted to improve annotation consistency. Annotations completed by one annotator were independently checked by another annotator. Cases involving disagreement in bounding-box placement or behavioral-category assignment were jointly reviewed by the three annotators until a consensus was reached. Instances with severe occlusion, insufficient visibility, or ambiguous behavioral states were marked as invalid and excluded from the final annotation set. The consensus annotations were subsequently used as the ground-truth labels for training, validating, and testing the classroom behavior detection models.

Faster R-CNN, Dynamic R-CNN, and Cascade R-CNN were compared to determine the detector used for classroom behavior extraction. Faster R-CNN was used as a conventional two-stage object-detection baseline [27]. Dynamic R-CNN dynamically adjusts training thresholds and regression parameters according to proposal quality [28], whereas Cascade R-CNN progressively refines object localization through a sequence of detectors trained with increasing intersection-over-union thresholds.

Detection model training. To ensure a fair comparison, Faster R-CNN, Dynamic R-CNN, and Cascade R-CNN were trained using the same implementation environment and core optimization settings.The experimental configuration followed the setup adopted in a closely related post-secondary classroom small-object-detection study. All models were implemented in Python 3.8 using PyTorch 1.8.1 and trained under Ubuntu 18.04 on an NVIDIA RTX 3080 Ti GPU. The backbone networks were initialized using ImageNet-pretrained weights. Stochastic gradient descent was used as the optimizer, with an initial learning rate of 0.002, a batch size of 8, and 400 training epochs. A warm-up learning-rate strategy was applied during the initial stage of training. Except for their architecture-specific components, the three detectors used the same training configuration to ensure comparability.

To reduce leakage caused by visually adjacent frames, the images were partitioned at the classroom-recording-session level into training, validation, and test sets using an approximate ratio of 70:20:10. All frames extracted from the same recording session were assigned to the same subset. Detection performance was evaluated using ${\mathrm{mAP}}_{50}$ and ${\mathrm{mAP}}_{75}$, following established object-detection evaluation conventions [29].

Student identity linkage. For each classroom recording session, an anonymized seating map was established using attendance records and a clear reference frame collected at the beginning of the session. Each student identifier was associated with a predefined rectangular seating region. For each detected student, the center coordinates of the bounding box were normalized by the image width and height and compared with the centers of the predefined seating regions using Euclidean distance. A match was accepted only when the detected center was located within the corresponding seating region and its distance from the seating-region center did not exceed half of the normalized diagonal length of that region.

When temporary seat changes occurred, the session-specific seating map was manually updated using adjacent video frames and attendance information. Observations were treated as invalid when a student was absent, outside the effective camera view, severely occluded, or insufficiently visible, or when a detection could not be uniquely associated with a seating region. These observations were excluded from subsequent behavioral aggregation and were not assigned to the “other” behavioral category. Identity-linkage accuracy was evaluated through stratified manual verification covering all four courses and the early, middle, and late course stages. A total of 150 classroom images were sampled, and all valid student detections within these images were individually verified against attendance records and the corresponding classroom reference frames. As shown in Table 2, 4590 identity-linkage instances were reviewed, of which 4484 were correctly linked, yielding an overall accuracy of 97.7%.

After quality control, window-level observations were aggregated into student–week records. The nine-week observation period generated 3375 student–week records. For student *i* during week *w*, the proportions of looking toward the instructional area, lowered-head/head-down behavior, and other visible states were calculated as

(1) $$LF_{i,w}=\frac{N_{LF,i,w}}{N_{\mathrm{valid},i,w}},$$

(2) $$LD_{i,w}=\frac{N_{LD,i,w}}{N_{\mathrm{valid},i,w}},$$

(3) $$Other_{i,w}=\frac{N_{\mathrm{Other},i,w}}{N_{\mathrm{valid},i,w}},$$

where $N_{LF,i,w}$, $N_{LD,i,w}$, and $N_{\mathrm{Other},i,w}$ denote the numbers of valid observation windows assigned to the three behavioral categories, and $N_{\mathrm{valid},i,w}$ denotes the total number of valid windows for that student and week. Sampling windows in which a student was not reliably observable were therefore excluded from both the behavioral counts and $N_{\mathrm{valid},i,w}$.

After weekly aggregation, each student was represented by a sequence of LF and LD proportions over the available observation weeks. Because LF+LD+Other=1, the Other proportion was not included as an independent predictive feature. The resulting weekly LF/LD sequences served as the basis for the raw, temporal, and prototype-based behavioral representations described in Section 2.3. $N_{\mathrm{valid}}$ was used only in the calculation and quality control of weekly proportions and was not used as an additional weighting factor across weeks.

### 2.3. Outcome-Independent Behavioral Representation and Experimental Design

All classroom behavioral representations were constructed independently of the final academic outcome. The weekly LF and LD sequences were represented using four forms: Raw weekly features, Temporal descriptors, hard unsupervised behavioral profiles, and continuous prototype-distance features. Their construction and corresponding experimental configurations are summarized in Table 3.

The Raw representation directly concatenated the weekly LF and LD proportions available up to each prediction point. The Temporal representation provided a more compact description of the longitudinal sequences using three components: behavioral level, temporal trend, and variability. Behavioral level was represented by the mean LF/LD proportion within each completed three-week stage, temporal trend by the slope of a linear fit against week index, and variability by the standard deviation of the observations available up to the corresponding checkpoint. This resulted in 6, 10, and 14 Temporal features at Weeks 3, 6, and 9, respectively.

K-means clustering [30] was further applied to the standardized Temporal features to identify recurring longitudinal behavioral patterns without using academic-outcome labels. Candidate values K∈{2,3,4} were compared using the Silhouette coefficient [31], Calinski–Harabasz index [32], and Davies–Bouldin index [33]. Each student was assigned to the nearest learned centroid to obtain the Hard-profile representation, while the Euclidean distances to all centroids were retained as continuous Prototype-distance features. Feature scaling, cluster-number selection, K-means fitting, and centroid estimation were performed using training students only within each cross-validation fold.

The primary behavioral-representation experiment compared B0–B4 using the same downstream classifier and identical student-level folds. The Background baseline contained gender, major, and entrance score, whereas the regular assessment score was excluded so that the contribution of classroom behavior could be evaluated relative to information available before the course.

Checkpoint-specific prediction was conducted at Weeks 3, 6, and 9. The Week-3, Week-6, and Week-9 models used behavioral observations from Weeks 1–3, Weeks 1–6, and Weeks 1–9, respectively. Raw and Temporal representations were reconstructed at each checkpoint using only the information available by that week. Gender, major, and entrance score formed the baseline at all checkpoints, and regular assessment was excluded.

Finally, regular assessment was examined in a separate information-source fusion experiment because it became available during the course. Four configurations were evaluated: F0 (Background), F1 (Background + Behavior), F2 (Background + Regular assessment), and F3 (Background + Regular assessment + Behavior). The behavioral representation retained from the preceding comparison was used consistently in this analysis.

### 2.4. Prediction Models and Evaluation Protocol

The prediction target consisted of three predefined academic-performance categories derived from final-examination scores: At-risk (final-examination scores < 70), Good (70 ≤ final-examination scores < 85), and Excellent (final-examination scores ≥ 85). The final sample included 144 At-risk, 157 Good, and 74 Excellent students. These thresholds were defined a priori before model training to distinguish academically at-risk, satisfactory-performing, and high-performing students.

XGBoost was used as the fixed downstream classifier for the behavioral- representation comparison, checkpoint-specific prediction, and final information-source fusion experiments. After the Temporal representation was retained from the behavioral-representation comparison, Logistic Regression, Random Forest [34], XGBoost [35], and LightGBM [36] were additionally compared using the same Temporal features and student-level folds to evaluate classifier robustness. This comparison was used to assess whether the predictive contribution of classroom behavior depended on a particular classifier rather than to select a different classifier for the subsequent fusion experiment. All predictive experiments used stratified five-fold cross-validation at the student level, with the same fold assignments used across comparable configurations. All data-dependent preprocessing and feature transformations were fitted using the training students only and then applied to the held-out students. For the prototype-based representations, feature scaling, cluster-number selection, K-means fitting, and centroid estimation were also performed exclusively within the training portion of each cross-validation fold [37]. Final academic-outcome labels were used only for training and evaluating the downstream classifiers and were not used in weekly behavioral aggregation, Temporal feature construction, or unsupervised behavioral-profile generation.

Performance was evaluated using Accuracy, Macro Precision, Macro Recall, Macro F1, and At-risk Recall [38]. Macro F1 was used as the primary overall metric, and at-risk recall was reported separately to evaluate the identification of academically at-risk students.

## 3. Results

### 3.1. Classroom Behavior Detection Performance

**Figure 1:**
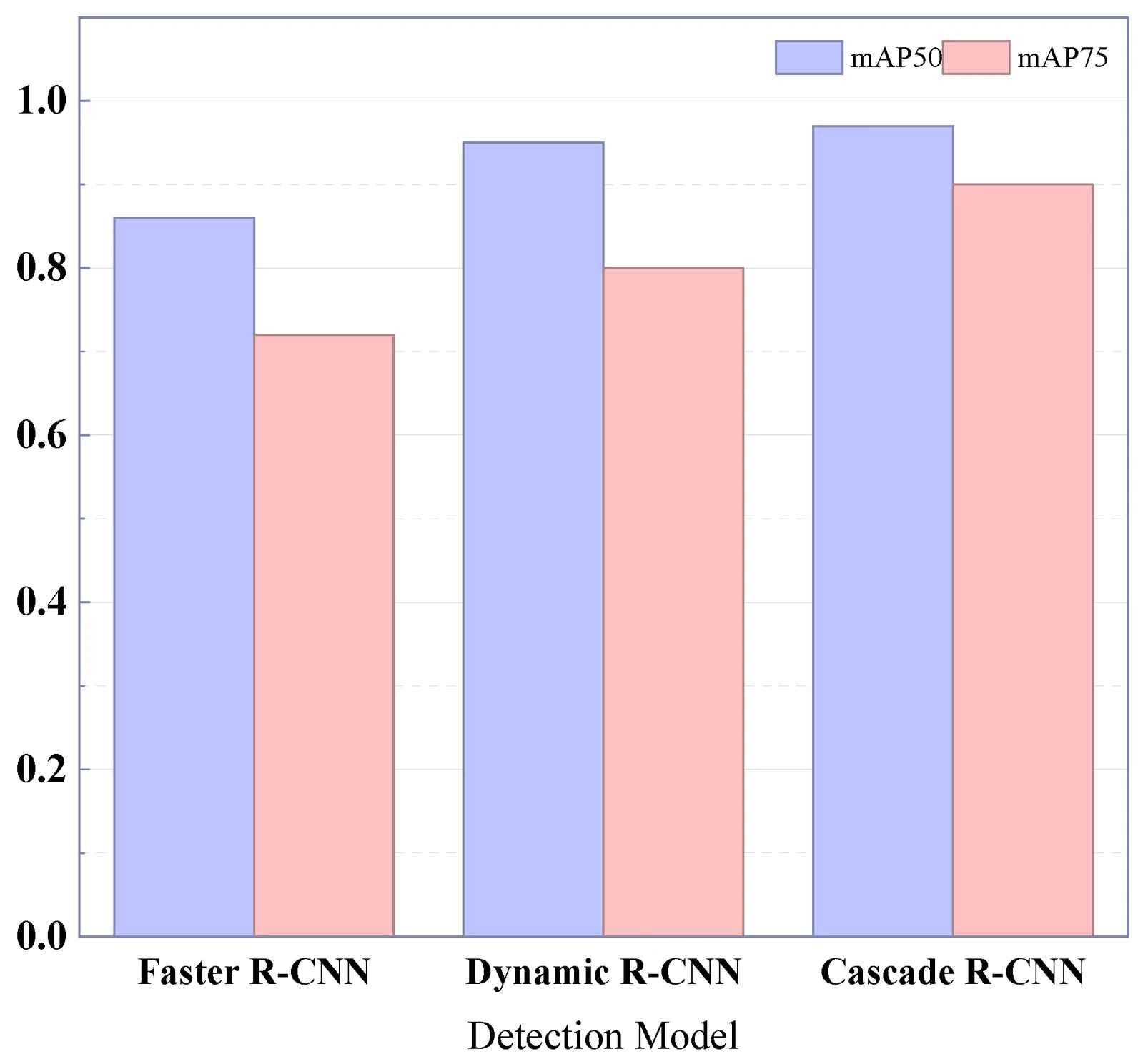
Performance comparison of Faster R-CNN, Dynamic R-CNN, and Cascade R-CNN in terms of ${\mathrm{mAP}}_{50}$ and ${\mathrm{mAP}}_{75}$.

The three object-detection models were compared to determine the detector used to construct the longitudinal classroom behavior records. As shown in Figure 1, Faster R-CNN achieved an ${\mathrm{mAP}}_{50}$ of approximately 0.86 and an ${\mathrm{mAP}}_{75}$ of 0.72. Dynamic R-CNN improved these values to approximately 0.95 and 0.80, respectively. Cascade R-CNN achieved the strongest overall detection performance, with an ${\mathrm{mAP}}_{50}$ of 0.97 and an ${\mathrm{mAP}}_{75}$ of 0.90.

The performance advantage of Cascade R-CNN was more pronounced under the stricter localization criterion. Its ${\mathrm{mAP}}_{75}$ exceeded that of Faster R-CNN by 0.18 and that of Dynamic R-CNN by 0.10. These results indicate that Cascade R-CNN produced more accurately localized detections in densely populated classroom scenes. It was therefore selected to generate the behavioral observations used in the subsequent longitudinal behavioral representation and academic-performance prediction analyses.

**Figure 2:**
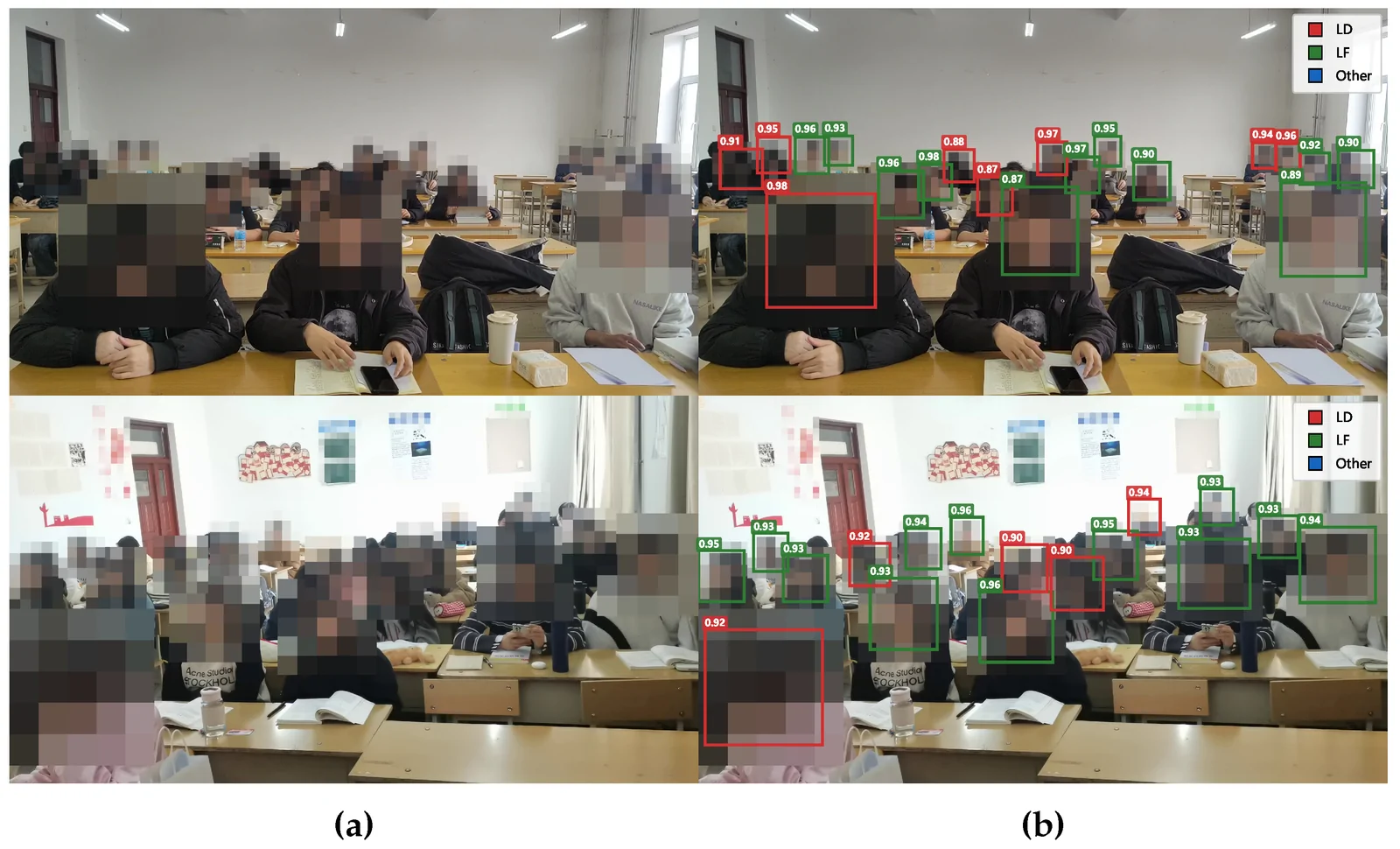
Representative classroom behavior detection results obtained using Cascade R-CNN from different fixed-camera viewpoints: (**a**) anonymized original classroom scenes and (**b**) corresponding behavior detection outputs.

Figure 2 presents representative detection examples obtained using Cascade R-CNN. The model localized students under variations in seating distance, classroom density, and partial occlusion and assigned the predefined observable behavior categories to the detected regions. The visualization provides a qualitative complement to the quantitative detection metrics rather than an independent assessment of the educational meaning of the detected behaviors. These examples are illustrative and do not constitute a separate evaluation of camera-viewpoint effects.

### 3.2. Unsupervised Longitudinal Behavioral Profiles

To characterize longitudinal classroom behavioral patterns without using academic-outcome labels, K-means solutions with K=2, K=3, and K=4 were evaluated using the full-sample Temporal descriptors for descriptive analysis. As shown in Table 4, the K=2 solution achieved the highest Silhouette coefficient (0.407) and Calinski–Harabasz index (310.07), together with the lowest Davies–Bouldin index (1.015). The corresponding values were 0.257, 243.55, and 1.309 for K=3, and 0.191, 189.54, and 1.568 for K=4. The K=2 solution was therefore retained for the descriptive characterization of longitudinal behavioral trajectories.

**Figure 3:**
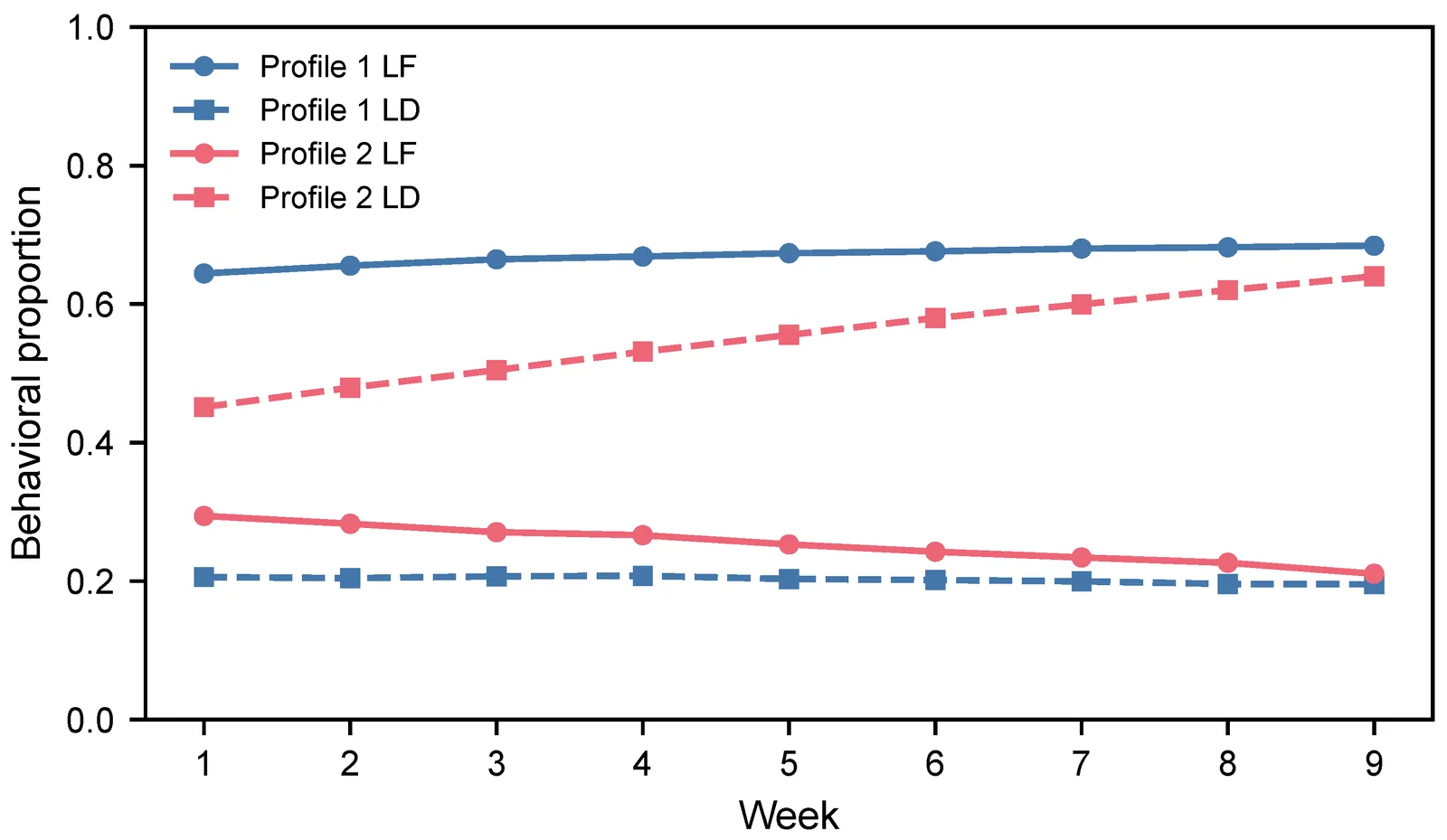
Weekly LF and LD trajectories of the two descriptive longitudinal behavioral profiles identified from the full sample using K-means clustering with K=2: High-LF profile (n=261) and Declining-LF profile (n=114). Lines represent weekly mean behavioral proportions.

The two descriptive longitudinal behavioral profiles obtained with K=2 are shown in Figure 3. The High-LF profile included 261 students (69.6% of the sample). Within this profile, the mean LF proportion remained comparatively high and increased slightly from 0.644 in Week 1 to 0.684 in Week 9, while LD remained low, changing from 0.206 to 0.195 over the same period. The Declining-LF profile comprised 114 students (30.4%). Its mean LF proportion decreased from 0.294 in Week 1 to 0.211 in Week 9, whereas LD increased from 0.451 to 0.640. The two profiles therefore exhibited clearly different longitudinal patterns in LF level and temporal direction, accompanied by contrasting changes in LD.

The full-sample clustering reported above was used only for descriptive characterization and visualization of the longitudinal behavioral patterns and was not used directly to construct predictive features. For the B3 and B4 predictive representations, feature scaling, cluster-number selection, K-means fitting, and centroid estimation were performed separately using the training students within each cross-validation fold. The K=2 solution was independently selected in all five training folds, consistent with the two-profile structure observed in the full-sample descriptive analysis.

The profile names High-LF and Declining-LF are descriptive labels for the observed behavioral trajectories and should not be interpreted as direct measures of attention, engagement, motivation, or learning quality. The predictive value of hard profile membership and continuous prototype-distance representations was examined in the subsequent behavioral-representation ablation.

### 3.3. Behavioral Representation Ablation

**Figure 4:**
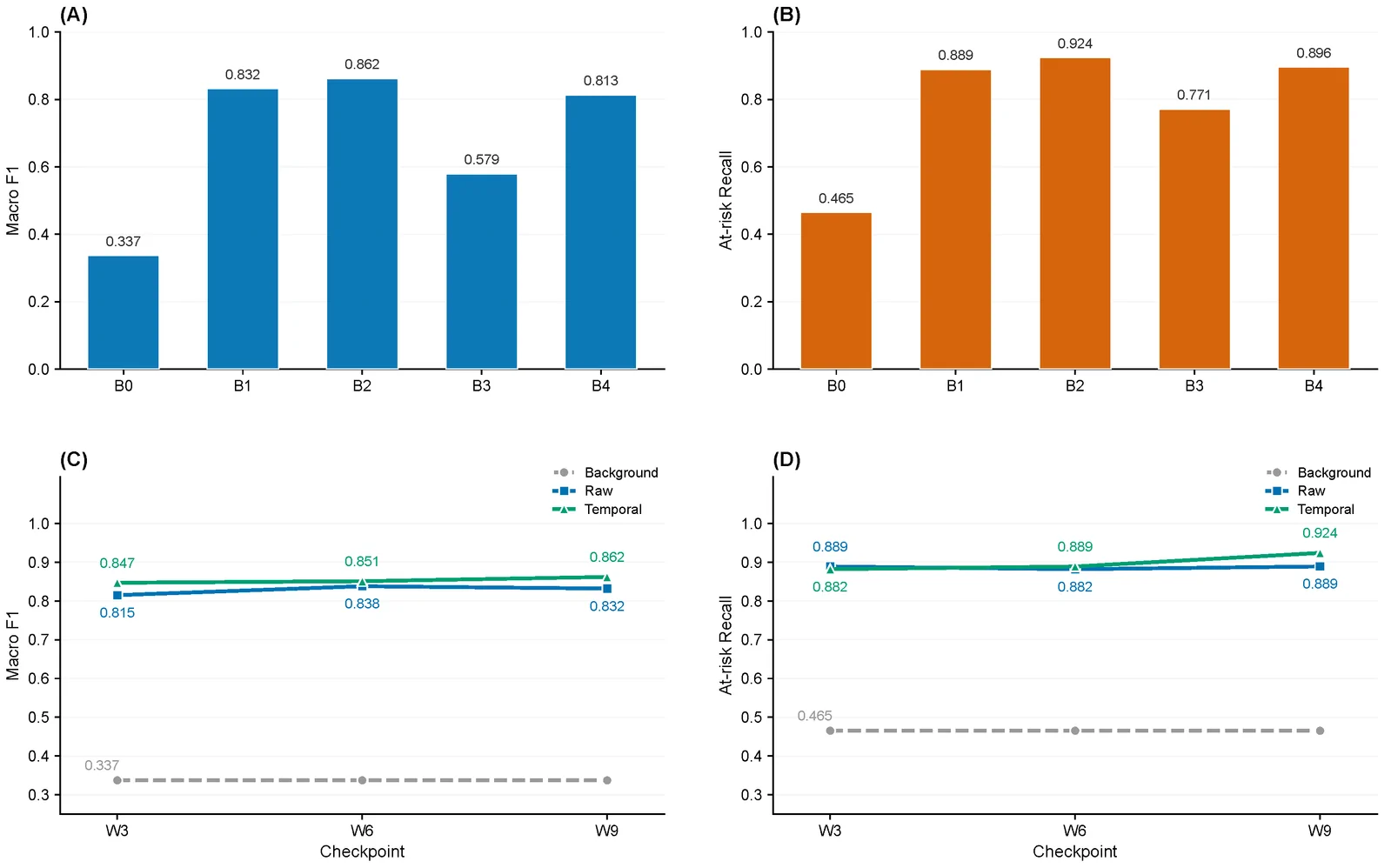
Behavioral-representation ablation and checkpoint-specific prediction performance: (**A**) Macro F1 for the B0–B4 behavioral-representation configurations; (**B**) At-risk Recall for the B0–B4 behavioral-representation configurations; (**C**) checkpoint-specific Macro F1 for the Background, Raw, and Temporal configurations at Weeks 3, 6, and 9; (**D**) checkpoint-specific At-risk Recall for the Background, Raw, and Temporal configurations at Weeks 3, 6, and 9.

To compare the predictive value of alternative representations of longitudinal classroom behavior, the five predefined B0–B4 configurations were evaluated using identical student-level cross-validation folds and the same downstream classifier. As shown in Figure 4, the Background-only configuration (B0) achieved a Macro F1 of 0.337 and an At-risk Recall of 0.465. Adding the Raw weekly LF/LD sequences substantially improved performance, with B1 achieving a Macro F1 of 0.832 and an At-risk Recall of 0.889. The Temporal representation (B2) achieved the strongest overall performance, with a Macro F1 of 0.862 and an At-risk Recall of 0.924.

The clustering-based representations also improved prediction relative to the Background baseline. B3, which represented each student using hard behavioral-profile membership, achieved a Macro F1 of 0.579 and an At-risk Recall of 0.771. Replacing the hard assignment with continuous distances to the learned behavioral prototypes substantially improved performance: B4 achieved a Macro F1 of 0.813 and an At-risk Recall of 0.896. This result indicates that continuous prototype-distance features retained more predictive information than a single hard profile assignment, although their performance remained below that of the Temporal representation.

Overall, the representation-level comparison showed that longitudinal classroom behavior provided substantial predictive information beyond the Background baseline. Among the evaluated representations, the Temporal features provided the strongest overall performance, while the Raw representation achieved closely comparable results. The Temporal representation was therefore retained as the primary behavioral representation for the subsequent classifier-robustness and information-source fusion analyses, whereas both Raw and Temporal representations were further evaluated in the checkpoint-specific analysis.

### 3.4. Checkpoint-Specific Prediction

Checkpoint-specific prediction was conducted at Weeks 3, 6, and 9 to examine how the predictive value of classroom behavior changed as additional longitudinal observations became available. Figure 4C,D compares the Background, Background + Raw, and Background + Temporal configurations at the three checkpoints.

Because the Background configuration contained only pre-course variables, its performance remained unchanged across checkpoints, with a Macro F1 of 0.337 and an At-risk Recall of 0.465. By Week 3, incorporating classroom behavior already produced a substantial improvement over this baseline. The Raw representation achieved a Macro F1 of 0.815 and an At-risk Recall of 0.889, while the Temporal representation achieved corresponding values of 0.847 and 0.882.

At Week 6, Raw achieved a Macro F1 of 0.838 and an At-risk Recall of 0.882, whereas Temporal achieved 0.851 and 0.889, respectively. With the complete nine-week behavioral history available, Temporal reached its strongest performance, with a Macro F1 of 0.862 and an At-risk Recall of 0.924. The corresponding Raw values were 0.832 and 0.889.

Overall, both behavioral representations consistently outperformed the Background baseline at all three checkpoints. The Week-3 results indicate that useful predictive information was already present in the classroom behavior observed during the early course stage. Temporal features showed a modest increase in Macro F1 as additional weeks became available and achieved their highest performance at Week 9, whereas Raw performance remained comparatively stable across the three checkpoints. These results support the use of longitudinal classroom behavior for time-bounded academic-performance prediction without requiring behavioral observations from later course stages.

### 3.5. Classifier Robustness Analysis

After the Temporal representation was identified as the strongest behavioral representation in the preceding ablation experiment, Logistic Regression, Random Forest, XGBoost, and LightGBM were compared using the same Temporal features and identical student-level cross-validation folds.

**Figure 5:**
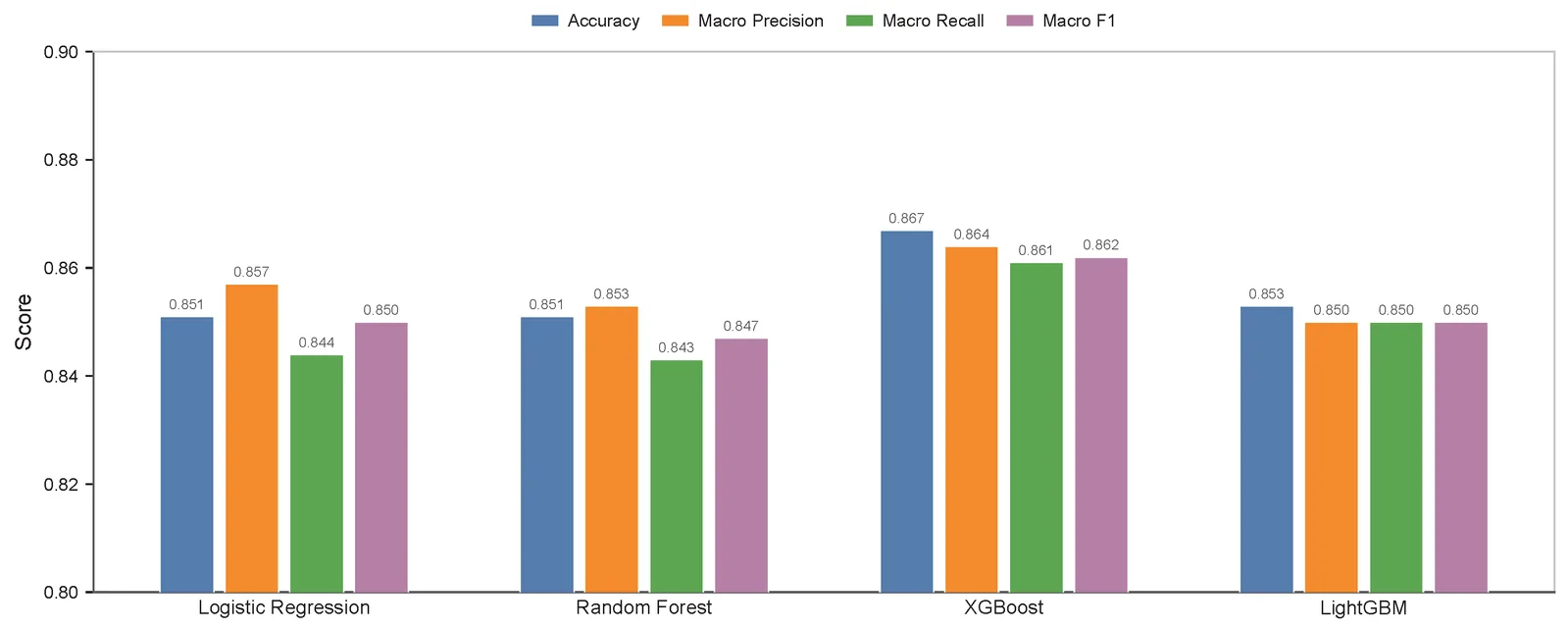
Predictive performance of Logistic Regression, Random Forest, XGBoost, and LightGBM using the Temporal behavioral representation. Accuracy, Macro Precision, Macro Recall, and Macro F1 are reported for each classifier under identical student-level cross-validation folds.

As shown in Figure 5, all four classifiers achieved broadly consistent performance using the Temporal behavioral representation. Logistic Regression achieved an Accuracy of 0.851, a Macro Precision of 0.857, a Macro Recall of 0.844, and a Macro F1 of 0.850. Random Forest achieved corresponding values of 0.851, 0.853, 0.843, and 0.847. XGBoost achieved the strongest overall performance, with an Accuracy of 0.867, a Macro Precision of 0.864, a Macro Recall of 0.861, and a Macro F1 of 0.862. LightGBM achieved corresponding values of 0.853, 0.850, 0.850, and 0.850.

Overall, the Temporal behavioral representation maintained strong predictive performance across all four classifiers, indicating that its predictive contribution was not specific to a single classification algorithm. XGBoost achieved the strongest overall performance, while the classifier comparison was treated as a robustness analysis rather than as a separate model-selection step. XGBoost remained the fixed downstream classifier for the subsequent information-source fusion experiment.

### 3.6. Information-Source Fusion Analysis

The final experiment evaluated the predictive contributions of longitudinal classroom behavior and regular assessment information using the fixed XGBoost classifier. Four information-source configurations were compared: F0 (Background), F1 (Background + Temporal), F2 (Background + Regular Assessment), and F3 (Background + Regular Assessment + Temporal).

As shown in Table 5, the Background-only configuration (F0) achieved an Accuracy of 0.403, a Macro F1 of 0.337, and an At-risk Recall of 0.465. Adding the Temporal behavioral representation (F1) increased these values to 0.867, 0.862, and 0.924, respectively.

Adding Regular Assessment to the Background variables (F2) also improved prediction relative to F0, achieving an Accuracy of 0.587, a Macro F1 of 0.552, and an At-risk Recall of 0.512. However, its performance remained considerably below that of F1, indicating that the longitudinal Temporal behavioral representation provided substantially stronger predictive information than Regular Assessment when each source was added separately to the same Background baseline.

Combining Background, Regular Assessment, and Temporal behavior (F3) produced the strongest overall performance, with an Accuracy of 0.896 and a Macro F1 of 0.895. Compared with F1, the addition of Regular Assessment further increased Accuracy from 0.867 to 0.896 and Macro F1 from 0.862 to 0.895, while At-risk Recall remained unchanged at 0.924.

Overall, both Regular Assessment and longitudinal classroom behavior provided predictive information beyond the pre-course Background variables, but the Temporal behavioral representation contributed the larger improvement when the two information sources were considered separately. Their combination in F3 yielded the strongest overall classification performance, supporting the complementary value of combining longitudinal classroom behavior with conventional educational information.

## 4. Discussion

The behavioral-representation ablation showed that the Temporal representation achieved the strongest overall predictive performance, while Raw weekly LF/LD features and Prototype-distance features also substantially outperformed the Background baseline.

The improvement over the Background baseline supports the complementary value of repeated classroom observations. The Background variables characterized student information available before the course, whereas the camera-derived features captured repeated externally observable classroom behavior. Previous research has shown that machine-learning methods can transform classroom recordings into structured behavioral indicators. The present study extends this work by demonstrating that longitudinal behavioral representations can contribute to academic-outcome prediction rather than serving only as behavior-recognition outputs. This finding is consistent with process-oriented learning analytics, in which behavioral evidence complements rather than replaces conventional academic information. Educational records and classroom behavior should therefore be regarded as related but non-equivalent sources of evidence.

The comparison of behavioral representations further showed that preserving longitudinal structure was important. The Temporal representation achieved the strongest overall performance, while Raw and Prototype-distance representations also retained substantial predictive information. Hard-profile membership performed considerably worse than the continuous Prototype-distance representation, suggesting that reducing each student to a single categorical profile discarded information that was partly retained by continuous distances to the learned behavioral prototypes. However, these results should not be interpreted as establishing the universal superiority of a particular representation across educational contexts. This cautious interpretation is consistent with multimodal learning-analytics research emphasizing that sensor-derived indicators should be interpreted through appropriate theoretical and contextual frameworks rather than treated as direct representations of latent learning constructs [39].

The checkpoint analyses showed that classroom behavior was already informative at Week 3 and remained predictive at Weeks 6 and 9. Temporal features showed a modest improvement as additional behavioral observations became available, whereas Raw performance remained comparatively stable across checkpoints. This finding is consistent with previous research showing that temporally organized learning traces can support early course-performance prediction. Related research on undergraduate early-warning systems has likewise shown that useful predictive performance can be obtained before course completion, highlighting the practical value of evaluating prediction models at intermediate course checkpoints [40]. The classifier-robustness analysis further showed that the predictive value of the Temporal representation was maintained across Logistic Regression, Random Forest, XGBoost, and LightGBM, with XGBoost achieving the strongest overall performance. This suggests that the contribution of classroom behavior was not restricted to a single classification algorithm.

The final information-source fusion analysis provided additional evidence of the complementary role of classroom behavior. Temporal behavioral features substantially improved prediction over the Background baseline. Regular Assessment alone improved predictive performance relative to the Background baseline, although the improvement was considerably smaller than that obtained by incorporating the Temporal behavioral representation. However, combining Regular Assessment with Temporal behavior increased Macro F1 from 0.862 to 0.895 and Accuracy from 0.867 to 0.896, while At-risk Recall remained unchanged at 0.924. This pattern suggests that regular assessment provided additional information for overall class discrimination once combined with longitudinal behavior, but did not further improve the identification of At-risk students. High detection accuracy alone would not establish educational relevance; the improvement over the Background baseline provides more direct evidence that the extracted trajectories added predictive information. Nevertheless, educational records and behavioral representations retain different meanings. Their integration should be interpreted as complementary data fusion rather than as multiple measurements of a single construct such as engagement.

The behavioral representations were constructed without using final academic-performance labels, and data-dependent preprocessing and clustering were fitted using training students only within each cross-validation fold. Accordingly, the reported results support an incremental predictive association between observable classroom behavior and academic performance rather than a causal effect of particular behaviors on achievement.

STEAP should therefore be viewed as a retrospectively evaluated, time-bounded predictive framework rather than as an already deployed automated early-warning system. Practical deployment would also require prospective validation, probability calibration, and evaluation of intervention effects. Because visible classroom behavior is context-dependent, model outputs should supplement instructor judgment rather than support automatic judgments of student ability or learning quality [41]. A lowered head, for example, may reflect reading or note-taking rather than disengagement. Recent human-centred learning-analytics research similarly emphasizes the importance of stakeholder involvement, human control, safety, reliability, and trustworthiness when educational analytics and AI systems are translated into practice [42].

Several limitations should be acknowledged. First, the sample was drawn from four computing-related courses at one university, and the results primarily represent internal validation; broader evaluation across semesters, institutions, courses, and instructional settings is needed. Second, the behavioral taxonomy was based primarily on observable head-orientation and head-posture cues sampled at 30 s intervals. These indicators do not comprehensively represent students’ classroom behavior and should not be interpreted as direct measures of attention, engagement, cognition, motivation, emotion, or learning quality. In addition, because the observed sessions were primarily conducted under conventional seated classroom conditions, the present behavioral observations may not adequately characterize movement-intensive activities such as laboratory work, student presentations, or other activities requiring students to leave their seats. Third, the smaller Excellent group may have reduced the stability of class-specific predictive estimates. Fourth, the use of demographic variables, educational records, and classroom video requires continued attention to fairness, privacy, data governance, and appropriate educational use. These concerns are particularly important in multimodal learning analytics, where the collection and combination of student data can introduce tensions between analytical value, privacy, and potential data encroachment [43].

## 5. Conclusions

This study proposed STEAP, a camera-based static–temporal data-fusion framework for academic performance prediction in computing and software engineering education. The framework transformed classroom videos into student-level weekly behavioral sequences and constructed outcome-independent longitudinal representations, including Raw weekly features, temporal descriptors, and unsupervised behavioral-profile representations. These behavioral features were integrated with educational information to evaluate their incremental predictive value and the resulting predictive performance at Weeks 3, 6, and 9.

The results showed that longitudinal classroom behavior provided substantial predictive information beyond the pre-course Background baseline. In the behavioral-representation comparison, the Temporal representation achieved the strongest performance, with a Macro F1 of 0.862 and an At-risk Recall of 0.924, compared with 0.337 and 0.465 for the Background-only baseline. Useful predictive information was already present at the Week-3 checkpoint, and the Temporal representation reached its strongest performance with the complete nine-week behavioral history.

The classifier-robustness analysis showed that this predictive contribution was maintained across multiple classification algorithms, with XGBoost achieving the strongest overall performance. In the final information-source fusion experiment, combining Background, Regular Assessment, and Temporal behavior produced an Accuracy of 0.896 and a Macro F1 of 0.895. These results support the complementary predictive value of longitudinal classroom behavior and indicate that its integration with conventional educational information can further improve overall academic-performance prediction.

## Figures and Tables

**Table 1:** Educational information.

Variable	Type	Domain	Brief description	Availability
Gender	Categorical	Student background	Student gender information	Before the course
Major	Categorical	Student background	Software Engineering or Applied Statistics	Before the course
Entrance score	Continuous	Prior academic achievement	College entrance examination score reflecting prior academic preparation	Before the course
Regular assessment score	Continuous	Course performance	Regular assessment score reflecting course-related academic performance	During the course; used only in final classification

**Table 2:** Manual verification results for student identity linkage.

Course Stage	Sampled Images	Identity-Linkage Instances	Correctly Linked Instances	Accuracy (%)
Early (Weeks 1–3)	50	1520	1475	97.0
Middle (Weeks 4–6)	50	1580	1548	98.0
Late (Weeks 7–9)	50	1490	1461	98.1
Overall	150	4590	4484	97.7

**Table 3:** Outcome-independent behavioral representations and feature configurations used in the primary comparison.

ID	Configuration	Behavioral Representation	Behavioral Dimension
B0	Background	No behavioral features; gender, major, and entrance score formed the pre-course baseline.	0
B1	Background + Raw	Weekly LF and LD proportions concatenated in chronological order.	6/12/18 at W3/W6/W9
B2	Background + Temporal	Stage-level LF/LD means and linear trends, together with cumulative LF/LD variability.	6/10/14 at W3/W6/W9
B3	Background + Hard profile	Nearest K-means behavioral prototype derived from the Temporal features.	1 profile feature
B4	Background + Prototype distance	Euclidean distances from the Temporal representation to the learned behavioral prototypes.	*K* distance features

**Table 4:** Internal clustering-validity results for the full-sample descriptive analysis. Upward arrows indicate that higher values are preferred, whereas the downward arrow indicates that lower values are preferred.

*K*	Silhouette ↑	CH ↑	DB ↓
2	0.407	310.07	1.015
3	0.257	243.55	1.309
4	0.191	189.54	1.568

**Table 5:** Information-source fusion performance using the fixed XGBoost classifier.

Configuration	Accuracy	Macro F1	At-Risk Recall
F0 Background	0.403	0.337	0.465
F1 Background + Temporal	0.867	0.862	0.924
F2 Background + Regular Assessment	0.587	0.552	0.512
F3 Background + Regular Assessment + Temporal	0.896	0.895	0.924

## Data Availability

The datasets generated and analyzed during the current study are not publicly available due to privacy and confidentiality considerations. For academic research purposes, anonymized derived data and supporting code may be requested from the corresponding authors, subject to institutional approval and applicable data-use requirements.
